# Studying Pregnancy Outcome Risk in Patients with Systemic Lupus Erythematosus Based on Cluster Analysis

**DOI:** 10.1155/2023/3668689

**Published:** 2023-01-27

**Authors:** Arezou Bikdeli, Daqing Li, Minati Malide, Meysam Nouri, Hongsheng Sun, Qingrui Yang, Naser Golsanami, Dongxia Liu

**Affiliations:** ^1^Key Laboratory of Cardiovascular Remodeling and Function Research, Chinese Ministry of Education, Chinese National Health Commission and Chinese Academy of Medical Sciences, State and Shandong Province Joint Key Laboratory of Translational Cardiovascular Medicine, Department of Cardiology, Qilu Hospital, Cheeloo College of Medicine, Shandong University, Jinan, 250012 Shandong, China; ^2^School of Basic Medical Sciences, Cheeloo College of Medicine, Shandong University, Jinan, 250012 Shandong, China; ^3^Department of Rheumatology and Immunology, Shandong Provincial Hospital Affiliated to Shandong First Medical University, Jinan 250021, China; ^4^School of Engineering, Shandong University of Science and Technology, Qingdao, 266590 Shandong, China

## Abstract

**Background:**

Pregnancy in systemic lupus erythematosus (SLE) patients is a challenge due to the potential maternal and fetal complications. Therefore, a multidisciplinary assessment of disease risk before and during pregnancy is essential to improve pregnancy outcomes.

**Objectives:**

Our purpose was to (i) define clusters of patients with similar history and laboratory features and determine the associative maternal and perinatal outcomes and (ii) evaluate the risk spectrum of maternal and perinatal outcomes of pregnancy in SLE patients, represented by our established risk-assessment chart.

**Methods:**

Medical records of 119 patients in China were analyzed retrospectively. Significant variables with *p* < 0.05 were selected. The self-organizing map was used for clustering the data based on historical background and laboratory features.

**Results:**

Clustering was conducted using 21 maternal and perinatal features. Five clusters were recognized, and their prominent maternal manifestations were as follows: cluster 1 (including 27.73% of all patients): preeclampsia and lupus nephritis; cluster 2 (22.69%): oligohydramnios, uterus scar, and femoral head necrosis; cluster 3 (13.45%): upper respiratory tract infection; cluster 4 (15.97%): premature membrane rupture; and cluster 5 (20.17%): no problem.

**Conclusion:**

Pregnancy outcomes in SLE women fell into three categories, namely high risk, moderate risk, and low risk. Present manifestations, besides the medical records, are a potential assessment means for better management of pregnant SLE patients.

## 1. Introduction

Systemic lupus erythematosus (SLE) is an autoimmune disease, which is principally found in women, especially in reproductive age. Pregnancy is considered high risk due to a combination of maternal risks (lupus flare, diabetes, and preeclampsia) and fetal risks (miscarriage, intrauterine fetal demise, preterm birth, intrauterine growth restriction, and congenital heart block) [1–3]. Considering the simultaneous involvement of both mother and fetus, it is always the patient's and the physician's concern to know the disease status and changes as well as evaluate pregnancy risks either before conception during six-month control or throughout the pregnancy. Such a fact indicates the importance of a planned pregnancy which can lead to the most favorable maternal-fetal outcomes [4] and is a protective approach against undesired pregnancy complications. For this purpose, counseling and risk assessment of the SLE patients should be considered before conception for evaluating poor pregnancy outcome, discussing contraindications of pregnancy, organ evaluation, and appropriately modifying medicine. During and after pregnancy, the cooperation of obstetrician and rheumatologist should be continued as a team in the planned visits. The best pregnancy outcome will be achieved in patients who are in remission 6 months before conception [2]. Nevertheless, in a patient with a new-onset or diagnosed in pregnancy, it is rather difficult to achieve this goal. Patients with high risk due to the flare of disease should postpone pregnancy until the disease is well-control. With this in mind, the need for risk stratification has always been a major concern of the researchers and physicians [5–10]. The unmet medical need that the present study aimed at addressing is the risk stratification for pregnancy outcomes by considering both maternal and perinatal outcomes, which remain as a major rheumatological and obstetric challenge.

Researchers have tried different techniques to investigate risk factors for adverse pregnancy outcomes [11] and have examined the values of angiogenic factors in early gestation [12]. Logistic regression has been used for estimating fetal loss in SLE women [13], and the Chi-square test has been adopted to evaluate maternal and fetal outcomes [14]. A meta-analysis of the most recent studies (2017-2019) has investigated maternal and fetal complications associated with SLE to update our knowledge of the present situation [15]. Artificial intelligence has also been implemented in this field to establish clinical decision support systems (CDSS) [9, 16, 17] and also to predict the probability of stillbirth or live birth [9]. Deep learning has been implemented for predicting lupus hospitalization and lupus hospital readmission, estimating extreme preterm birth, and evaluation of pathological observations in Glomerular lupus nephritis [18–21]. Moreover, clustering has been used for evaluating the spectrum of serum autoantibodies in pediatric-onset SLE [22], investigating the relationship between damage and mortality rate in the juvenile-onset of lupus disease [23], characterizing lupus patients profiles [24, 25], and also studying patients with antiphospholipid syndrome [26–29]. However, evaluating the outcome risk of pregnancy in SLE patients has not been studied using neural network clustering. The artificial intelligence approach has proved more powerful than [30–32] or as powerful as statistical methods [33] in dealing with complex medical problems.

In the present study, we also addressed the issue of mixed-type data in clinical studies. Clinical data would be expressed as categorical (nominal and ordinal) and quantitative (continuous, or discrete numbers) values [34]. For each individual kind of these data, there are well-established statistical analysis methods, which are convenient for straightforward usage by physicians. However, how to use two or several kinds of these data together is not simple. The data we had in the present study included both categorical (low, high, and normal) and continuous kinds. Therefore, we employed the concepts of “cooccurrence” and “similarity of phenomena” through a particular algorithm to find the continuous equivalent of a categorical item. Then, the two values would be compared and appropriately used by any statistical or mathematical model for such tasks as dimension reduction, clustering, and classification. For convenient usage of the reader, a step-by-step guide is provided for this algorithm in the supplementary materials (available here).

Therefore, in this study, the primary objective was to detect clusters of SLE women based on their historical background and laboratory features. Then, we determined the association of these clusters with the risk of maternal and perinatal outcomes.

## 2. Patients and Method

This was a single-center retrospective study with cross-sectional analysis. We clustered the patients based on their historical background and laboratory features. After obtaining clusters, the corresponding maternal manifestations and perinatal outcomes of each cluster were identified, and the clusters were characterized. Then, the obtained clusters were divided into three groups, namely the high-, medium-, and low-risk groups. Finally, to display the disease status, a risk-assessment chart was designed.

### 2.1. Patients

Medical records of 119 pregnant SLE patients from 2015 to 2019 in Qilu Hospital of Shandong University in Jinan city, Shandong Province of China, were reviewed. The present study was exempt from Institutional Review Board approval (IRB) as it was merely a clinical analysis of pregnant SLE women. It did not include any human experimentation or application of any new drugs or experiments on the tissues of the patients. The patients were all women with a primary or secondary diagnosis of SLE approved by at least two rheumatologists. Herein, 16% of the patients were diagnosed with SLE during pregnancy, and the rest were all diagnosed before pregnancy. The selection criteria of the patients included (i) women with approved pregnancy, (ii) no limitation for the age and disease duration, and (iii) met the American College of Rheumatology (ACR) 1997 revised criteria for SLE [35, 36]. We had no specific criteria for the exclusion of the patients. Information about the neonatal APGAR and neonatal weight as well as the type of delivery was obtained from the delivery room. The pregnant SLE patients included both multipara and nullipara. Although we had no age limit in the selection criteria, the patients were 19 to 43 years old, for whom the gestational age ranged from 35 to 290 days. The average maternal age and gestational age were, respectively, 29.23 years old and 237.65 days. We identified 22 potential variables in this regard; however, one of these variables, CH 50, was not appropriately recorded for all of the patients. Hence, we had to leave it out.

### 2.2. Data

Three researchers (the first, third, and fourth authors) extracted the variables' data from the medical records at Qilu Hospital of Shandong University. In the next step, a professional monitor with experience in rheumatologic studies reviewed the extracted data and identified inconsistencies, which were corrected after reconsideration and discussion with other team members. The research team included four experts in the field of Rheumatology and Immunology, two experts in the field of Gynecology and Obstetrics, and two experts in the field of data science and corresponding data analysis of the present study. With regard to pregnancy outcomes, we, respectively, had 15 maternal outcomes and four perinatal outcomes. The maternal outcomes were items *y*_1_-*y*_15_ (represented in the next section), and the perinatal outcomes were live birth/fetal loss, term/preterm, newborn's weight, and first-minute Apgar. The adopted variables included disease duration, mother's age at pregnancy, gestational age at delivery, type of delivery, and obstetric historical background of the mother (GPAL). Our serological features included antinuclear antibodies (ANA), anti-double-stranded DNA (dsDNA) antibodies, antiphospholipid antibodies (APS), anti-Ro/SSA, and anti-La/SSB. Complement C3 and C4 were also measured. Baseline routine laboratory tests included WBC, RBC, PLT, HB, random urine test, erythrocyte sedimentation rate (ESR), and C-reactive protein (CRP). Our recorded data included both the numerical and categorical types (mixed-type data). While disease duration, mother's age, urinary protein, gestational age at delivery, newborn weight, and GPAL were numerical data, type of delivery, HB, PLT, RBC, WBC, C3, C4, ESR, ANA, CRP, SSB Ab, SSA Ab, dsDNA, and APS were categorical data. The categories were labeled “low,” “normal,” and “high.” Moreover, urinary protein variations fell in the ranges of negative, trace (less than 10 mg/dl), 1+ (30 mg/dl), 2+ (100 mg/dl), and 3+ (300 mg/dl), 4+ (1000 mg/dl) [37, 38]. Besides to new onset of proteinuria, the kidney activity of the patients had been confirmed by the presence of decreasing complement levels, rising anti-double-stranded DNA, and the new onset of hypertension [39]. Preterm labor was defined as “born before 37 complete weeks of gestation” [40], while term labor was defined as “born at a gestational age between 37 and 42 weeks.” The Apgar score was used to assess neonatal vitality, including the items of appearance, pulse, grimace, activity, and respiration, with total scores ranging from 0 to 10. The definition of stillbirth was fetal death at or after 20 weeks' gestation [41], and miscarriage was defined as loss of pregnancy before 20 weeks of gestation. It should be noted that we encountered some missing values in the extracted data which is to be expected in such a retrospective study. The missing data were for SSA and SSB (each, four patients), APS (seven patients), and dsDNA and ANA (each, five patients). For the purpose of consistency and quality of the employed data, we tried to complete this data before clustering. The issue was resolved by replacing the mode of each class for categorical variables and the mean of each class for numerical variables. This approach is well-established and has been reported by previous research studies as well [9, 42, 43].

### 2.3. Joint Application of Numerical and Categorical Values

**Figure 1:**
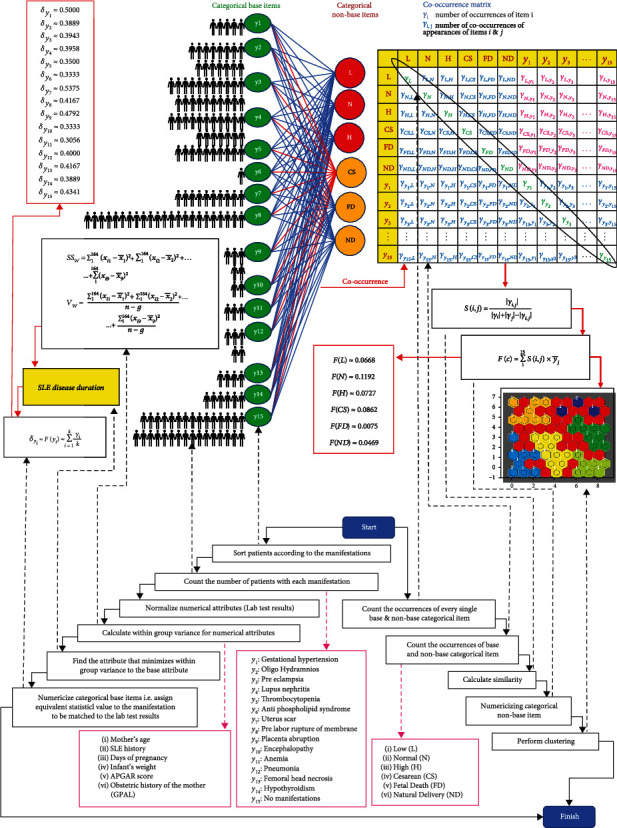
Adopted methodology for the joint usage of categorical and numerical data.

Considering that our data was mixed-type, based on the concept of cooccurrence, we implemented the algorithm of “two-step method for clustering mixed numerical and categorical data (TMCM).” [44] TMCM starts with selecting a particular characteristic (base attribute) in the categorical data and compares it with other characteristics (nonbase attributes). While cooccurrences (similarity) of the base and nonbase attributes were calculated using Equation (1), the within-group sum (*SS*_*w*_) of squares and within-group variance (*V*_*w*_) were, respectively, calculated using Equations (2) and (3).

(1) $$\begin{array}{c} S\left(i,j\right)=\frac{\left|O\left(i,j\right)\right|}{\left|O\left(i\right)\right|+\left|O\left(j\right)\right|-\left|O\left(i,j\right)\right|}, \end{array}$$

where *O*(*i*, *j*) represents the cooccurrence or similarity between *i*th nonbase item with the *j*th base items and *O*(*i*) and *O*(*j*), respectively, represent the total number of occurrences of each nonbase and base items.

(2) $$\begin{array}{c} {SS}_{w}=\overset{119}{\underset{1}{\sum }}{\left(x_{i1}-\bar{x_{1}}\right)}^{2}+\overset{119}{\underset{1}{\sum }}{\left(x_{i2}-\bar{x_{2}}\right)}^{2}+\cdots +\overset{119}{\underset{1}{\sum }}{\left(x_{i9}-\bar{x_{9}}\right)}^{2}, \end{array}$$

(3) $$\begin{array}{c} V_{w}=\frac{\sum_{1}^{119}{\left(x_{i1}-\bar{x_{1}}\right)}^{2}+\sum_{1}^{119}{\left(x_{i2}-\bar{x_{2}}\right)}^{2}+\cdots +\sum_{1}^{119}{\left(x_{i9}-\bar{x_{9}}\right)}^{2}}{n-g}, \end{array}$$

where *x*_*i*1_ to *x*_*i*9_ indicate the nine numerical attributes, *n* stands for the number of observations, i.e., patients, and *g* is the number of numerical attributes. It was possible to quantify the nonbase categorical items using the following equation:

(4) $$\begin{array}{c} F\left(c\right)=\overset{15}{\underset{1}{\sum }}S\left(i,j\right)\times \bar{y_{j}}, \end{array}$$

where *c* represents the categorical nonbase item (*c*_1_ to *c*_6_), *S*(*i*, *j*) is the similarity between the *i*th nonbase item and the *j*th base item *y*(*y*_1_ to *y*_15_).  Table 1 indicates the observed similarity between the base and nonbase variables of the current research. The categorical labels for different variables are represented in Table 2. The adopted methodology is depicted in Figure 1.

### 2.4. Adopted Self-Organizing Network

Neural networks' superior performance in healthcare studies has been proven by many research works [9, 45, 46]. The advent of deep learning in recent years has even further highlighted the significance and prospective applications of neural networks in various directions of medical studies. Deep learning and the feature engineering techniques have enabled obtaining deeper insight into the data that were not easily obtainable earlier [47, 48]. These networks are the simple mathematical formulation of the superfast and overcomplicated learning process which occurs in the human brain [49, 50]. The human information processing system is composed of neurons switching at speeds about a million times faster than the logical computer gates [51]. The knowledge in the artificial neural network is not stored in a single neuron but inside all the neurons and their meaningful (weighted) connections with the neighboring neurons [52]. The current study adopted the clustering capability of neural networks through the self-organizing maps (SOM). Clustering is the most typical form of unsupervised learning and is a crucial application of machine learning. However, its potential has not been fully explored in medical studies. Self-organizing maps are used to reduce the dimensionality so as to better understand the data in hand. SOM networks are one of the most important components of artificial neural networks and are very sensitive to the input values as all other machine learning algorithms. Therefore, in the present study, appropriate preprocessing of the input variables was required. We first observed the data and removed the potential outliers. Since values of different variables had different min-max ranges, we normalized all input variables to prevent the impact of these ranges. The normalization was applied as:

(5) $$\begin{array}{c} x_{normal}=\frac{x-x_{\min}}{x_{\max}-x_{\min}}. \end{array}$$

**Figure 2:**
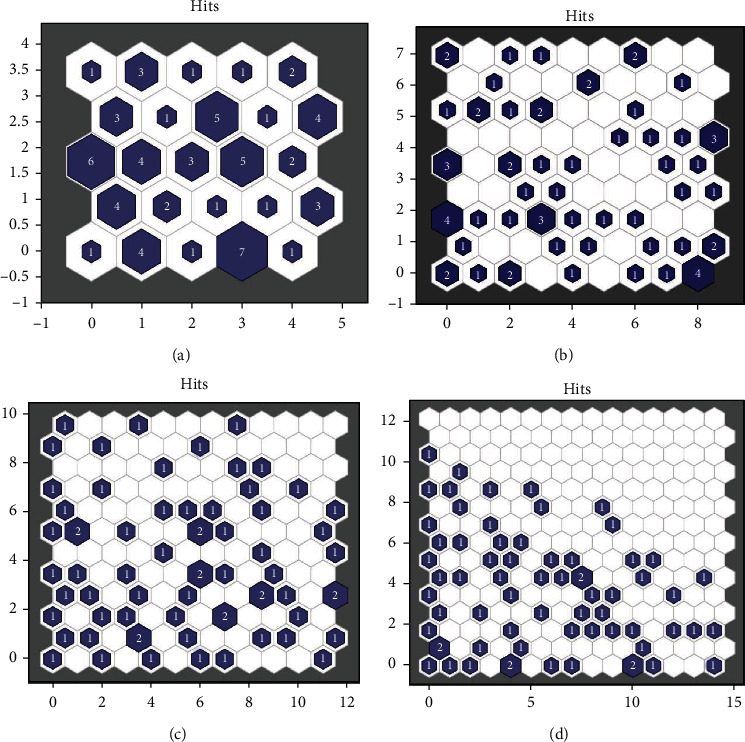
SOM sample hits. The hits are for clustering the patients using (a) 5, (b) 9, (c) 12, and (d) 15 neurons in the hidden layer. Five and eight n4eurons are insufficient, while 15 neurons are more than necessary.

**Figure 3:**
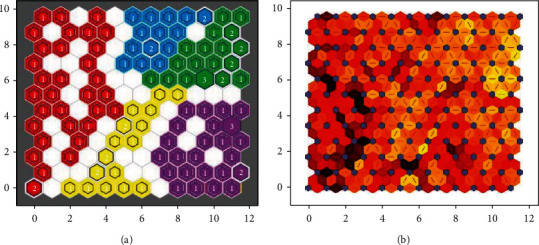
(a) Clustering results where the patients fell into five clusters. (b) Neighbor weight distances. A darker color indicates a larger distance, which means a greater difference between the corresponding data point of any two neurons.

In the established self-organizing maps of the present study, the optimal network structure and the number of neurons in the hidden layer were selected based on the trial-and-error method and were confirmed by the researchers' experience of the field. This is a well-known approach in the application of machine learning algorithms [9, 17, 53, 54]. Herein, different hidden layers, respectively, using 5, 9, 12, and 15 neurons were examined (Figures 2(a)–2(d)). Eventually, the best-performing network had a 1-1-1 structure with one input layer, one hidden layer, and one output layer. It was observed that 12 neurons could satisfyingly capture the information among the input characteristics of the patients. Our SOM was trained in the batch mode using “trainbu” function (learnsomb). In batch training, the network's weight and biases are updated at the end of an entire pass through the input data [55]. The network performance function was mean square error. By selecting 12 neurons in the hidden layer, a topology consisting of 12 × 12 = 144 miniclusters was constructed where each neuron acted as an independent minicluster center. Then, those miniclusters which were close enough to each other were collected together to form a high-level cluster. These final clusters are depicted in Figure 3(a). The idea of subclusters and high-level clusters is explained in detail in the research work of Vesanto and Alhoniemi [56].

## 3. Results and Cluster Analyses

Our patients were divided into five clusters. Figure 3(a) shows these clusters on the self-organizing map, and Figure 3(b) represents the distance between the adjacent neurons of the SOM.

**Figure 4:**
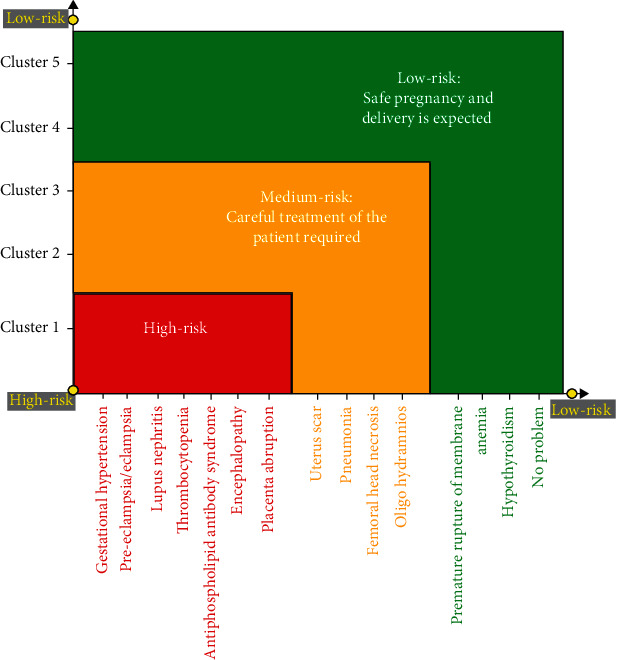
Proposed risk-assessment chart of the present study for evaluating the disease status and changes.

Cluster 1 included 33 patients for whom the maternal manifestations were mainly preeclampsia/eclampsia and lupus nephritis. We had 21 patients with preeclampsia/eclampsia among which 8 patients had eclampsia. The perinatal outcomes included 6 miscarriages (5.8%), 3 of which were with LN, 1 with preeclampsia, and 2 with a spontaneous abortion without any manifestation. A total of 15 fetal deaths (11.76%) included 4 with preeclampsia, 3 with gestational hypertension, 3 with lupus nephritis, 1 with AP antibody syndrome, 2 with encephalopathy, and 2 with thrombocytopenia. The cause of encephalopathy in the patients was intracranial hypertension (IH). IH is a poorly understood disorder characterized by increased intracranial pressure usually idiopathic in the absence of an identifiable lesion. It is a rare manifestation of lupus in pregnancy [57].The average APGAR was <7, and the average newborn weight was 950 g. Patients in this cluster were the “most high risk.” In our proposed risk-assessment chart for predicting the disease status and changes (Figure 4), this cluster is shown in red color. It is worth noting that 3 patients had antiphospholipid syndrome (anticardiolipin antibodies) which were in clusters 1 (2 patients) and 2 (1 patient).

Clusters 2 and 3, with 27 and 16 patients, respectively, included a better pregnancy outcome with a few cases of those maternal manifestations in cluster 1. Oligohydramnios and uterus scar were the prominent maternal manifestations found in these two clusters. Upper respiratory tract infection, placenta abruption, encephalopathy, and femoral head necrosis were also observed in a few cases. We identified the risk of these two clusters as “moderate.” The average APGAR was between 9 and 10, and the average newborn weights were 2800 g and 2400 g, respectively. This group is shown with yellow in the risk-prediction chart.

Clusters 4 and 5 were the “low-risk pregnancy” clusters which included 19 and 24 patients, respectively. Cluster 4 was identified with preterm rupture of membrane (PROM) as the prominent maternal manifestations. Cluster 5 was mainly characterized by no damage. Anemia and hypothyroidism were the common manifestations of these two clusters. The average APGAR was almost 10 (9.94 and 9.91, respectively), and the average newborn weights were 2850 g and 2950 g, respectively. This group is shown with green in the risk-prediction chart.

Tables 3 and 4 denote the detailed analysis of the resultant clusters.

It should be noted that Figure 3(b) shows the internal structure of the SOM topology. In this figure, each node contains the data (patients) with the most similar characteristics. The variables of the patients could not be individually interpreted from this figure but the overall differences between any two patients could be inferred. In the self-organizing maps, the neurons are in a competitive structure, which learns to recognize groups of similar input vectors in such a way that neurons physically near each other in the neuron layer respond to similar inputs [55]. This means that for a clustering task using SOMs, one could not use cutpoints to categorize the continuous variables and assign each individual patient to a particular cluster. This is because we are dealing with a high number of variables (more than three) where the human mind is incapable of analyzing all the cutpoints and the entangled relationships of those variables. This is the most important reason for using the dimension reduction technique as in the present study. In SOMs, while a group of similar and dissimilar variables coexists, differentiating between two similar ones is not feasible but differentiating between a similar and dissimilar one is quite accessible. In the context of this study, this means that our model could not discriminate, e.g., between lupus nephritis and preeclampsia, because both manifestations indicate a high-risk pregnancy outcome. This issue is an unmet medical need as well. Meanwhile, our method differentiates among the patients in different clusters based on their input characteristics for risk stratification. If two patients are different in their input data and pregnancy outcomes and fall into two different clusters, then our methods could differentiate between lupus activity and infection; but if two different manifestations are assigned to the same cluster, such a differentiation is not feasible.

## 4. Discussion

In this retrospective research of pregnant SLE patients, we observed several patterns in the history and laboratory features and analyzed their association with different pregnancy outcomes. Pregnancy outcome in SLE women is often influenced by not a single but several factors to different degrees. Hence, we applied the clustering technique which is the first study to conduct such an analysis in pregnant SLE patients. To the best of our knowledge, the advantage of identifying these clusters compared to the identification of poor or good prognostic factors demonstrated in the daily clinical practice of patients lies in the fact that the clustering method enables us to analyze numerous factors simultaneously through dimensionality reduction. Since these factors have different degrees of influence on pregnancy outcome risk, self-organizing maps could capture the relationship between these factors. Usually, there is no obvious linear relationship between influencing factors and the pregnancy outcome, which adds to the complexity of the problem [58–62]. Previous studies on pregnant SLE patients have focused only on a very limited number of these factors (e.g. [41, 63, 64]).

Five distinct clusters were identified based on the history and laboratory features, which were then explored for their association with maternal manifestation and perinatal outcomes. We found considerable differences among obtained clusters not only in the proportion of associative maternal manifestations but also in the perinatal outcomes, both of which determine the risk of pregnancy outcome. Tables 5 and 6 represent the detailed analysis of the mother's and infant's features in the obtained clusters.

**Figure 5:**
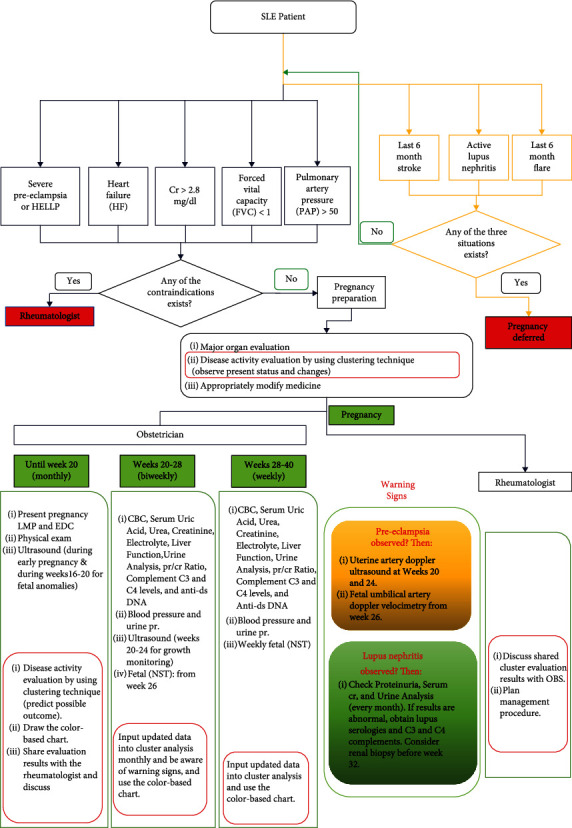
Management protocol of lupus patients during different stages of pregnancy. The red boxes indicate those sections that follow our introduced methodology.

Based on the obtained information, a risk-assessment chart was proposed for evaluating the disease risk (Figure 4). This chart, which is specially designed for pregnancy, includes most of the possible maternal manifestations in pregnant SLE patients and demonstrates their corresponding probable perinatal outcomes. Moreover, for clarifying when to use the introduced methodology of the present study, a management protocol is introduced (Figure 5). Nevertheless, the detailed analysis of the obtained clusters revealed that cluster 1 was the largest and the only one to include stillbirth and abortion (21 cases, 17.6%) while all the patients in other clusters had a live birth. In the studied patients, no maternal mortality was observed. In clusters 2 and 3, the most observed fetal outcome was preterm (19 cases, 15.9%). Clusters 4 and 5 were the low-risk clusters, and the born babies were mostly full term (32 cases, 26.8%). All of these five clusters differed significantly in their predominant maternal manifestations. Patients with preeclampsia/eclampsia and LN mainly represented cluster 1, while those with PROM damage were mainly in cluster 4. Cluster 5 was the one that included the highest number of no-damage patients. When considering all clusters together, we also observed that pregnant patients without problems were the most common, i.e., 22 patients (18.48%), preeclampsia was the second common manifestation with 21 patients (17.64%), and lupus nephritis and premature rupture of membrane, each with 17 patients (14.28%) were the third common manifestations. There was a good agreement between each cluster's manifestations severity and the number of live and death births, Apgar score, and the newborn weight. This observation verified the reliability of the clustering results. Besides, based on the perinatal outcomes (live/fetal death, term/preterm birth, newborn weight, and first-minute APGAR) and maternal manifestations, we found that pregnant SLE patients could have three levels of disease risk, i.e., high risk, moderate risk, and low risk. In our research, 27.73%, 36.13%, and 36.13% of the patients were, respectively, at high risk, moderate risk, and low risk for pregnancy. Totally, 39 labors (32.7%) were preterm, and 59 (49.5%) labors were term. We found preterm delivery in 7.5% of high-risk patients, in 15.9% of moderate-risk patients, and in 9.2% of low-risk patients. The obtained results of the present study were in agreement with those of other researchers, which verifies the reliability of the adopted approach. As also follows from these studies, the patients at the high-risk category, with predominantly preeclampsia/eclampsia and LN, are associated with an increased risk of stillbirth and abortion and the risk for cesarean section. Moreover, the risk of newborns who had low birth weight, and newborns with an APGAR score < 7 within 1 minute were significantly associated with SLE [6, 15, 53]. Therefore, cluster 1 which belonged to the high-risk category was the best predictor for fetal death. This cluster was also the best predictor for maternal complications, which in detail were gestational hypertension, preeclampsia/eclampsia, lupus nephritis, thrombocytopenia, AP antibody syndrome, encephalopathy, and placenta abruption. Patients in the moderate-risk category had more chance of preterm delivery; and for the low-risk category, which included no damage and PROM, it would be a full-term baby. Surprisingly, PROM which is usually considered an emergency and needs a quick decision fell in the low-risk category in the present study. The reasons for this phenomenon included the following: (i) We had 17 patients (0.71%) with PROM while only five of them were preterm PROM (0.29%). Most of the fetuses born due to PROM were mature at 37-40 weeks without complications. Hence, most of the newborns were term birth without fetal death, with average 1 minute APGAR of 9.82, and the mean newborns' weights were 3145 g for PROM and 2570 g for preterm PROM. The existing studies have also indicated that among women with lupus, spontaneous preterm labor has not proved to drive the high preterm birth rate [54]. (ii) In our target study center, induction of labor is more favorable than expectant management which may lead to a lower risk of maternal infection. Moreover, as reported in the previous studies [2], and also, in a single-center case series, researchers observed that preterm PROM was common in SLE pregnancy while it was not associated with disease activity.

According to our results, rheumatologist and obstetrician should bear the high-risk group in mind which shows a high mortality rate in pregnant SLE patients. The previous studies have recommended frequent antenatal visits before 20 gestational weeks in high-risk SLE patients [22]. Moreover, our proposed protocol (Figure 5) which includes screening lupus patients before conception and follow-up visits during pregnancy would be useful for the proper management of SLE patients. Herein, the information about disease status and changes could be obtained as follows. Physicians could follow the protocol in their visits at the designated times. Then, they could implement our introduced methodology to determine associative maternal and fetal outcomes and see the relevant changes of the disease condition by pointing and comparing its present and previous locations on the proposed risk-assessment chart (Figure 4).

In this research, we noticed that cluster 1, which was identified by the high-risk patients, included two patients with no problems. Moreover, cluster 4, which was recognized as the low-risk cluster, had two preeclampsia and two lupus nephritis patients. This implies that only considering manifestations and analyzing the patients based on their manifestations as conducted by the previous research studies could not come to reliable results. However, using the intelligent technique can display the hidden aspect of the patient s' data.

## 5. Conclusion

In conclusion, we put forth a new approach for identifying different patterns of history and laboratory features within pregnant SLE patients. We observed that the patients' pregnancy outcomes could be high risk, moderate risk, or low risk. Herein, our findings were neither manifestations nor lab results because either of these two is not individually enough to represent the patient's condition reliably. It is better to incorporate different medical records and optimize management procedures. Therefore, a well-planned pregnancy and risk stratification 6 months before pregnancy and continuous multidisciplinary assessments during pregnancy are necessary for SLE pregnant patients.

The application of neural networks, especially deep learning and function fitting tools, for predicting the level of SLE disease activity would be suggested as a topic for future studies.

## 6. Limitations and Future Research

The most notable limitations of the present study are as follow: (a) The adopted methods of imputation and substitution for making up for the missing data pose bias on the original data and could cause misinterpretation of the results. Therefore, the ratio of the mission data should be considered carefully. (b) Several adopted variables of this retrospective study (such as HB, RBC, WBC, ESR, C3, and C4) had been recorded as categorical items. However, continuous values are preferable because they cover a wider range and are more precise than the categorical values. (c) SLE disease activity should be measured and validated by disease activity instruments, such as SLEDAI and BILAG. The introduced methodology of the present study is not capable of producing such results. (d) The authors believe that a greater number of patients would certainly lead to more reliable results.

## Figures and Tables

**Table 1:** Similarity matrix representing the cooccurrence of different variables.

	L	N	H	CS	FD	ND	y_1_	y_2_	y_3_	y_4_	y_5_	y_6_	y_7_	y_8_	y_9_	y_10_	y_11_	y_12_	y_13_	y_14_	y_15_
L	259	0	0	0	0	0	4	18	52	49	20	15	7	24	9	3	7	8	5	12	26
N	0	893	0	0	0	0	35	83	133	90	18	2	21	154	21	22	11	32	15	33	223
H	0	0	395	0	0	0	13	29	88	82	14	22	11	43	9	14	8	12	6	7	37
CS	0	0	0	89	0	0	1	8	15	10	2	2	3	15	3	1	1	4	2	3	18
FD	0	0	0	0	22	0	3	1	6	6	2	1	0	0	0	2	0	0	0	0	1
ND	0	0	0	0	0	8	0	1	0	1	0	0	0	2	0	0	1	0	0	0	3
y_1_	0	0	0	0	0	0	4	0	0	0	0	0	0	0	0	0	0	0	0	0	0
y_2_	0	0	0	0	0	0	0	10	0	0	0	0	0	0	0	0	0	0	0	0	0
y_3_	0	0	0	0	0	0	0	0	21	0	0	0	0	0	0	0	0	0	0	0	0
y_4_	0	0	0	0	0	0	0	0	0	17	0	0	0	0	0	0	0	0	0	0	0
y_5_	0	0	0	0	0	0	0	0	0	0	4	0	0	0	0	0	0	0	0	0	0
y_6_	0	0	0	0	0	0	0	0	0	0	0	3	0	0	0	0	0	0	0	0	0
y_7_	0	0	0	0	0	0	0	0	0	0	0	0	3	0	0	0	0	0	0	0	0
y_8_	0	0	0	0	0	0	0	0	0	0	0	0	0	17	0	0	0	0	0	0	0
y_9_	0	0	0	0	0	0	0	0	0	0	0	0	0	0	3	0	0	0	0	0	0
y_10_	0	0	0	0	0	0	0	0	0	0	0	0	0	0	0	3	0	0	0	0	0
y_11_	0	0	0	0	0	0	0	0	0	0	0	0	0	0	0	0	2	0	0	0	0
y_12_	0	0	0	0	0	0	0	0	0	0	0	0	0	0	0	0	0	4	0	0	0
y_13_	0	0	0	0	0	0	0	0	0	0	0	0	0	0	0	0	0	0	2	0	0
y_14_	0	0	0	0	0	0	0	0	0	0	0	0	0	0	0	0	0	0	0	4	0
y_15_	0	0	0	0	0	0	0	0	0	0	0	0	0	0	0	0	0	0	0	0	22

The detailed explanation of the variables and labels are provided in Table 2.

**Table 2:** Adopted variables of the patients.

Index	Category
*Categorical nonbase items*	
L	Lower than standard
N	Normal value
H	High value
CS	Cesarean section
FD	Abortion or fetal death
ND	Natural delivery
*Categorical base items*	
*y*_1_	Gestational hypertension
*y*_2_	Oligohydramnios
*y*_3_	Preeclampsia
*y*_4_	Lupus nephritis
*y*_5_	Thrombocytopenia
*y*_6_	Antiphospholipid syndrome
*y*_7_	Uterus scar
*y*_8_	Prelabor rupture of membrane (PROM)
*y*_9_	Placenta abruption
*y*_10_	Encephalopathy
*y*_11_	Anemia
*y*_12_	Pneumonia
*y*_13_	Femoral head necrosis
*y*_14_	Hypothyroidism
*y*_15_	No manifestations
*Numerical attributes*	
*x*_1_	Disease duration (years)
*x*_2_	Mother's age (years)
*x*_3_	Urinary protein (mg/dl)
*x*_4_	Gestational age (days)
*x*_5_	Newborn's weight (kg)
*x*_6_	Gravid (G)
*x*_7_	Para (P)
*x*_8_	Abortion (A)
*x*_9_	Live (L)

**Table 3:** Analysis of the categorical laboratory features: No. of patients with each feature in the resultant clusters.

	Categorical values	DsDNA	ANA	APL	CRP	ESR	SSA	SSB	C3	C4	HB	PLT	RBC	WBC	Type of delivery	Total No. of patients
Cluster 1	1⁣^∗^						6	5	15	11	15	13	15	5	CS (12)	
	2⁣^∗^	9	5	20	19	10	12	23	17	20	17	20	18	14	FD (20)	33
	3⁣^∗^	23	28	13	14	23	15	5	1	1	1			14	ND (1)	

Cluster 2	1⁣^∗^		1				4	5	6	5	16	4	11	0	CS (22)	
	2⁣^∗^	18	9	23	17	13	15	9	21	22	11	23	16	16	FD (1)	27
	3⁣^∗^	9	17	4	10	14	8	3	0	0	0			11	ND (4)	

Cluster 3	1⁣^∗^						3	2	6	5	8	2	16	0	CS (15)	
	2⁣^∗^	5	3	13	10	4	7	13	9	10	8	14	10	9	FD (1)	16
	3⁣^∗^	11	13	3	6	12	6	1	1	1	0			7	ND (0)	

Cluster 4	1⁣^∗^						5	2	5	5	14	6	10	2	CS (17)	
	2⁣^∗^	8	8	18	10	9	9	16	9	13	5	13	9	13	FD (0)	19
	3⁣^∗^	11	11	1	9	10	5	1	5	1	0			4	ND (2)	

Cluster 5	1⁣^∗^							2	5	6	14	1	11	3	CS (23)	
	2⁣^∗^	19	8	24	17	12	17	21	19	16	10	21	13	13	FD (0)	24
	3⁣^∗^	5	16	0	7	12	7	1	0	2	0	2		8	ND (1)	

Footer: 1⁣^∗^: low, 2⁣^∗^: normal, and 3⁣^∗^: high.

**Table 4:** Analysis of mother's features: history, average fetal weight, APGAR, and numerical lab features.

	Maternal age at delivery time (years)	Disease duration (years)	Gestational age (days)	Gravid (G)	Para (P)	Abortion (A)	Live (L)	Preterm	Urinary protein	
									Value	No. of patients
Cluster 1	28.93	4.842	178.36	2	0.93	1.33	0.51	9 (7.5%)	Negative	7
									1+	5
									2+	8
									3+	11
									4+	2

Cluster 2	31.12	4.892	258.74	2.59	2	0.59	2	11 (9.2%)	Negative	14
									1+	5
									2+	4
									3+	4

Cluster 3	25.81	4.715	254	1.125	0.93	0.125	0.937	8 (6.72%)	Negative	4
									1+	2
									2+	5
									3+	5

Cluster 4	29.89	4.78	257.31	3.05	1.26	1.78	1.21	8 (6.72%)	Negative	11
									1+	3
									2+	5
									3+	0

Cluster 5	29.29	4.14	268.91	1	1	0	1.04	3 (2.5%)	Negative	18
									1+	6
									2+	0
									3+	0

**Table 5:** No. of patients with each manifestation observed in different clusters (mother's features).

	Cluster 1	Cluster 2	Cluster 3	Cluster 4	Cluster 5	Total	*p* value
Manifestation							
Gestational hypertension, *n* (%)	3 (2.5%)	1 (0.84%)	0	0	0	4	
Oligohydramnios	2 (1.6%)	3 (2.5%)	1 (0.84%)	1 (0.84)	3 (3.2%)	10	
Preeclampsia	10 (8.4%)	4 (3.3%)	5 (4.2%)	2 (1.6)	0	21	
Lupus nephritis	8 (6.7%)	3 (2.5%)	3 (3.2%)	3 (3.2%)	0	17	
Thrombocytopenia	2 (1.6%)	0	1 (0.84%)	0	1 (0.84%)	4	
Antiphospholipid syndrome	2 (1.6%)	1 (0.84%)	0	0	0	3	
Uterus scar	0	3 (2.5%)	0	0	0	3	
Prelabor rupture of membrane	1 (0.84%)	5 (4.2%)	2 (1.6%)	7 (5.8%)	2 (1.6%)	17	
Placenta abruption	1 (0.84%)	1 (0.84%)	0	1 (0.84)	0	3	
Encephalopathy	2 (1.6%)	0	1 (0.84%)	0	0	3	
Anemia	0	0	0	1 (0.84%)	1 (0.84%)	2	
Pneumonia	0	0	2 (1.6)	1 (0.84%)	1 (0.84%)	4	
Femoral head necrosis	0	1 (0.84%)	0	0	1 (0.84%)	2	
Hypothyroidism	0	0	0	1 (0.84%)	3 (2.5%)	4	
No manifestations	2 (1.6%)	5 (4.2%)	1 (0.84%)	2 (1.6%)	12 (10.8%)	22	
Variables (mean ± S.D.)							
Disease duration (years)	4.84 ± 4.86	4.89 ± 3.40	4.71 ± 4.27	4.79 ± 3.38	4.14 ± 3.73	4.69 ± 3.97	<0.005
Mother's age (years)	28.94 ± 4.51	31.11 ± 3.77	25.81 ± 4.37	29.90 ± 3.20	29.29 ± 2.07	29.23 ± 3.99	0.051
Gestational age (days)	178.40 ± 65.60	258.74 ± 17.11	254.00 ± 17.92	257.32 ± 14.63	268.92 ± 10.72	237.64 ± 52.05	<0.005
G	2.09 ± 0.98	2.59 ± 0.69	1.13 ± 0.34	3.05 ± 1.27	1.00 ± 0.00	2.01 ± 1.09	<0.005
P	0.94 ± 0.56	2.00 ± 0.00	0.94 ± 0.25	1.26 ± 0.45	1.00 ± 0.00	1.24 ± 0.55	<0.005
A	1.33 ± 0.99	0.59 ± 0.69	0.13 ± 0.34	1.79 ± 1.08	0.00 ± 0.00	0.81 ± 1.00	<0.005
L	0.52 ± 0.57	2.00 ± 0.28	0.94 ± 0.25	1.21 ± 0.42	1.04 ± 0.20	1.13 ± 0.66	<0.005

**Table 6:** No. of different perinatal outcomes in each cluster (infant's features).

Perinatal outcome	Cluster 1	Cluster 2	Cluster 3	Cluster 4	Cluster 5	Total
Live birth	12	27	16	19	24	
Fetal loss (death)	21					
Term birth	3	16	8	11	21	
Preterm birth	9	11	8	8	3	
Newborn's weight (mean ± S.D.)	952.00 ± 846	2818.00 ± 810	2419.00 ± 505	2823.00 ± 456	2952.00 ± 623	2274.70 ± 1086
First-minute Apgar	2.82 ± 3.96	9.59 ± 1.01	9.69 ± 0.87	9.95 ± 0.23	9.92 ± 0.41	7.85 ± 3.80

## Data Availability

The developed codes are included in the Appendix (Supplementary materials) of the present manuscript. Other data will be provided upon request.

## Supplementary Materials

Supplementary Materials S1 Quantifying categorical items. Table S1: Cooccurrence matrix of numerical and categorical variables. Figure S1: guideline chart on sorting the variables in the cooccurrence matrix. S2. Further details of the adopted neural network.
