# Supplementary material for: Studying Pregnancy Outcome Risk in Patients with Systemic Lupus Erythematosus Based on Cluster Analysis
Source: Biomed Res Int. 2023 Jan 27;2023:3668689. doi: 10.1155/2023/3668689 (PMC11521590; doi:10.1155/2023/3668689)
Supplement: Supplementary Materials — S1 Quantifying categorical items. Table S1: Cooccurrence matrix of numerical and categorical variables. Figure S1: guideline chart on sorting the variables in the cooccurrence matrix. S2. Further details of the adopted neural network. [file 3668689.f1.docx]

Supplementary Materials

## S1: Quantifying categorical items:

1. Consider the number of patients = k;

2. If the test result of a particular parameter (e.g. Disease Duration, Weigh, etc.) is a continuous number, it is considered a numerical attribute. Consider m = number of numerical attributes;

3. If the assessment result of a particular parameter (e.g. RBC, C3, etc.) is represented by categorical items such as low, high, normal, etc., the parameter is considered as a categorical attribute and each of the representative items is categorical items (non-base items). Consider n = number of non-base categorical attributes;

4. Each manifestation is considered as a categorical item (base-items);

5. Considering the preceding four steps, sort your data according to **Table A1**;

**Table S1** Co-occurrence matrix of numerical and categorical variables

|  | Categorical Non-Base Attributes | | | | | Categorical Base Attribute | Numerical Attributes | | | | |
| --- | --- | --- | --- | --- | --- | --- | --- | --- | --- | --- | --- |
| order | Param  (1) | Param  (2) | Param  (3) | … | Param  (n) | Manifestations | param  (1) | param  (2) | param  (3) | … | param  (m) |
| 1 |  |  |  |  |  |  |  |  |  |  |  |
| 2 |  |  |  |  |  |  |  |  |  |  |  |
| . |  |  |  |  |  |  |  |  |  |  |  |
| . |  |  |  |  |  |  |  |  |  |  |  |
| . |  |  |  |  |  |  |  |  |  |  |  |
| *k* |  |  |  |  |  |  |  |  |  |  |  |

6. Count the co-occurrence of categorical base and non-base attributes and sort them in a separate table. **Figure A1**is an example of such sorting as adopted in the present study and could be used as a typical example;


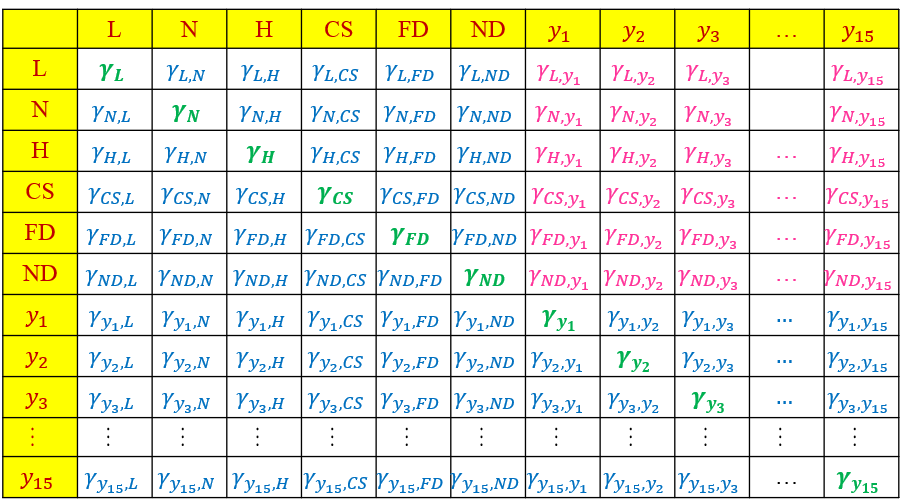


**Figure S1** Guideline chart on sorting the variables in the co-occurrence matrix

7. Follow the workflow in **Figure 1(**Adopted methodology for the joint usage of categorical and numerical data) of the present paper and find out the numerical value that corresponds to the occurrences of each of the categorical base and non-base items;

8. Create a matrix for patients’ data called “Data” where the number of rows is k, and number of columns is n+m+1;

9. Run the following typical code. This code is the typical example of the variables in the present research and could be modified at will for any number of variables;

for j=7:21 %ID of base items ( Based items are the items in the base attribute. If we consider manifestations as the base attribute, then each manifestation will be a base item). In the present study, we had 15 base items which stand in rows 7 to 21.

for i=1:6 %Number of categories (non-base attributes). Each non-base categorical attribute contained only one item. Hence we totally had six non-base items. Our six categories were L, N, H, CS, FD, ND.

D(i,j)=m(i,j)/(m(i,i)+m(j,j)-m(i,j)) % D is similarity matrix

end

end

for i=1:15 %Totally had 15 base items

Category1=sum((D(1,i)*Averages(1,i)))

Category2=sum((D(2,i)*Averages(1,i)))

Category3=sum((D(3,i)*Averages(1,i)));

Category4=sum((D(4,i)*Averages(1,i)))

Category5=sum((D(5,i)*Averages(1,i)))

Category6=sum((D(6,i)*Averages(1,i)))

end

10. Now the numerical values corresponding to categorical items are obtained. Together with numerical data we had from the beginning, these values will be used for clustering using the following code.

% Self-Organizing Map for clustering. This script assumes these variables are defined: Data: input data.

x = Data';

dimension1 = 12;% Creates a Self-Organizing Map

dimension2 = 12;

net = selforgmap([dimension1 dimension2]);

net.trainParam.epochs=200

% Choose Plot Functions. For a list of all plot functions type: help nnplot

net.plotFcns = {'plotsomtop','plotsomnc','plotsomnd','plotsomplanes', 'plotsomhits', 'plotsompos'}. ;

% Train, test, and view the Network, then create various plots

[net,tr] = train(net,x);

y = net(x);

view(net)

figure, plotsomtop(net)

figure, plotsomnc(net)

figure, plotsomnd(net)

figure, plotsomplanes(net)

figure, plotsomhits(net,x)

figure, plotsompos(net,x)

if (false)% Deployment. Change the (false) values to (true) to enable the following code blocks.

genFunction(net,'myNeuralNetworkFunction');% Generate a function for a neural network for application deployment

y = myNeuralNetworkFunction(x);

end

if (false)

genFunction(net,'myNeuralNetworkFunction','MatrixOnly','yes');

y = myNeuralNetworkFunction(x);

end

## S2: further details of the adopted neural network

SOM was first introduced by Kohonen in 1982 who described them as a biologically inspired approach to generate meaningful representations of data objects. Self-organization is a fundamental pattern recognition process, where essential inter-and intra-pattern relationships are learned without the presence of a potentially biased external influence. SOMs assign weights with the same dimensionality as the input data to each node in the primary low-dimensional map. Then, these weights are adjusted through the training process and the network finally creates regions on the primary map dependent on the structure of the input data. In the adopted neural network of the present study, two topologies, namely hexagonal and grid, and three distance functions, i.e., Euclidean distance, Manhattan distance, and link distance were explored. The network topology was chosen to be hexagonal with Euclidian distance between two neighboring neurons. As mentioned earlier, we used 12 neurons in the hidden layer of the implemented network. It should be noted that while a fewer number of neurons/layers would miss the meaningful relationship among the data (e.g., 5 neurons), an unreasonably large number of neurons and layers would also make the networks unnecessarily complex. Such complex networks usually have reduced generalization capability that is resulted from overfitting the existing data. The maximum number of epochs and maximum time to train the network were respectively set to 200 and infinity. The initial neighborhood size was 3, and the ordering phase steps were 100. Since neural networks start from a random initial weight matrix, retraining the SOM network would lead to different results. Nonetheless, to make the research methodology robust enough, we trained the network several times using the optimal network structure and found out that every time similar patients fell into the same clusters. Therefore, it was guaranteed that retraining would not question the reliability of our results.
